# On the Reliability of Examining Dual-Tasking Abilities Using a Novel E-Health Device—A Proof of Concept Study in Multiple Sclerosis

**DOI:** 10.3390/jcm9113423

**Published:** 2020-10-25

**Authors:** Niels Böttrich, Moritz Mückschel, Anja Dillenseger, Christoph Lange, Raimar Kern, Tjalf Ziemssen, Christian Beste

**Affiliations:** 1 MS Center Dresden, Centre for Clinical Neuroscience, Department of Neurology, Faculty of Medicine, TU Dresden, 01307 Dresden, Germany; niels.boettrich@gmail.com (N.B.); moritz.mueckschel@ukdd.de (M.M.); anja.dillenseger@ukdd.de (A.D.); tjalf.ziemssen@ukdd.de (T.Z.); 2 Cognitive Neurophysiology, Department of Child and Adolescent Psychiatry, Faculty of Medicine, TU Dresden, 01307 Dresden, Germany; 3 MedicalSyn GmbH, 01309 Dresden, Germany; christoph.lange@medicalsyn.com (C.L.); raimar.kern@medicalsyn.com (R.K.)

**Keywords:** multiple sclerosis, neuropsychology, assessment, dual-tasking, cognition, e-health, progressive web application

## Abstract

The assessment of neuropsychological functions and especially dual-tasking abilities is considered to be increasingly relevant in the assessment of neurological disease, and Multiple Sclerosis (MS) in particular. However, the assessment of dual-tasking abilities is hindered by specific software requirements and extensive testing times. We designed a novel e-health (progressive web application-based) device for the assessment of dual-tasking abilities usable in “bedside” and outpatient clinic settings and examined its reliability in a sample of N = 184 MS patients in an outpatient setting. Moreover, we examined the relevance of dual-tasking assessment using this device with respect to clinically relevant parameters in MS. We show that a meaningful assessment of dual-tasking is possible within 6 min and that the behavioral readouts overall show good reliability depending on dual-tasking difficulty. We show that dual-tasking readouts were correlated with clinically relevant parameters (e.g., EDSS, disease duration, processing speed) and were not affected by fatigue levels. We consider the tested dual-tasking assessment device suitable for routine clinical neuropsychological assessments of dual-tasking abilities. Future studies may further evaluate this test regarding its suitability in the long-term follow up assessments and to assess dual-tasking abilities in other neurological and psychiatric disorders.

## 1. Introduction

Cognitive dysfunctions are a frequent concomitant of Multiple Sclerosis (MS) with a prevalence of 40 to 70% [1,2]. The assessment of cognitive dysfunctions in MS is therefore central to the characterization and staging of the disease [1,3,4,5], which leads to the definition of specific MS phenotypes [6]. Several cognitive screening batteries have been developed. However, traditionally, the assessment of cognitive functions in MS is most centered around the examination of processing speed, attentional problems and memory problems, because these domains are among the first to show deficits in MS [4,7,8]. However, it has been argued that most of these neuropsychological assessments are limited, because they do no tap into abilities often referred to as “multitasking” [5], i.e., being able to cope with at least two demands at virtually the same time. Given that these abilities are important to cope with everyday life demands and other occupational requirements, a reliable assessment of these ability is highly desirable [5], and thus represent a largely unmet medical need for a holistic treatment of MS-patients. MS can be understood as a dysfunction of the human connectome [9] due to lesions in white matter structures [10,11,12]. Since large-scale networks have been shown to be important in dual-tasking [13,14,15,16,17,18,19,20,21,22,23], it seems reasonable that the assessment of dual-tasking abilities is essential in the neuropsychological assessment of MS. Yet, in the field of MS-research, dual-tasking has mostly been examined by assessing the performance of simple cognitive tasks while walking [24,25,26,27,28,29] or balancing [30,31]. These approaches, focusing on cognition-motor interactions [29], however, bear the problem that task difficulty is not parametrically scalable, and are thus possibly unsuitable to detect early dysfunctions and track disease progression [32]. Moreover, the assessment is not easily controllable [33].

To overcome these issues, a previous study by our group introduced the usage of dual-task to examine the above-mentioned abilities in MS patients [32]. This test is based on a so-called psychological refractory period (PRP) task. The PRP is a well-known phenomenon [34] and describes the finding that responses (RT2) on an external signal (stimulus) of a second task (S2) are slower or less accurate when this stimulus input is presented shortly after another (first) stimulus (S1), signaling a different reaction (RT1) (=PRP effect). Varying the time between S1 and S2, it is possible to scale the magnitude of the PRP effect and the difficulty of the task. With increasing time between S1 and S2, the PRP effect becomes smaller [35,36,37], because the response selection processes become less taxed [38,39,40]. We have shown [32] that MS patients performed considerably worse than healthy control participants and that deficits shown by the patients are very likely not due to simple motor deficits. Crucially, to date, the assessment of dual-tasking abilities using the PRP-test was time-consuming since test administration took about 30 min. More importantly, however, the administration of the test was difficult in outpatient settings because it required specific software packages and standard desktop PCs. Clinical usage and dissemination are strongly expedited if the test is short and can, ideally, be delivered flexibly—i.e., without specific software requirements in various settings. Therefore, the current study presents an e-health device allowing a PRP-based dual-tasking assessment using a tablet computer-based application which is already used in clinical practice [41]. We present data from a sample of N~200 MS patients and examine the reliability of dual-task assessment in this patient group, as well as the relation of dual-tasking performance using this tablet computer with clinically relevant parameters in MS.

## 2. Experimental Section

### 2.1. Patient Sample Description

In total, N = 206 MS patients were prospectively recruited at the MS Centre Dresden, University Hospital Carl Gustav Carus, Germany. The tests took place within the typical outpatient setting of the MS Centre Dresden. The study was approved by the local ethics committee. Written informed consent was obtained from all participants in accordance with the Declaration of Helsinki. The ethics committee of the TU Dresden approved the study.

Due to incomplete patient data regarding disease activity, and severe upper extremity motor disabilities, N = 22 patients were removed from the sample before data analysis was conducted. N = 184 patients (N = 130 female and N = 54 male) were included in the final analysis. The participants received no financial reimbursement for their participation. Participants were advised that participation, but also non-participation, in the study would not have any beneficial or detrimental effects on their patient care. The mean age was 42.04 (±10.8) years. School education was N = 11 patients with 9 years of school (corresponds to German “Hauptschulabschluss”), N = 78 patients with 10 years of school (German “Mittlere Reife”), N = 94 patients with at 12/13 years of school (German “Abitur”) or higher educational level. The premorbid intellectual performance (PIP) level was estimated for each patient, considering the level of school education, vocational training and professional requirements. N = 78 patients were classified as average PIP, N = 94 as above average and N = 10 below average PIP. The PIP was estimated on a custom (in-house) 8-point scale, with the following anchor points: A score of 1 refers to a substandard PIP, no school graduation and non-qualifying profession. A score of 4 was considered average PIP, 9-years of school education, vocational training and average professional requirements. A score of 5 is good average PIP, 10-years of school, vocational training and average professional requirements. A score of 6 refers to slightly above average PIP, 12/13 years of school, vocational training and average or slightly above average professional requirements. A score of 8 indicates above average PIP, university degree and high professional requirements. N = 175 participants were relapsing–remitting (RR) MS patients N = 5 secondary progressive (SP) MS and N = 4 primary progressive (PP) MS patients. N = 106 patients were classified as showing a moderate disease course, N = 78 an active disease course. For N = 171 patients, no disease activity (i.e., worsening of symptoms, relapses, MRI activity) was reported within the last 6 months, N = 8 patients showed activity within the last 2 months prior to participation. Medication was Ocrelizumab (N = 48), Dimethylfumarate (N = 30), Fingolimod (N = 25), Natalizumab (N = 19), Alemtuzumab (N = 16), Glatiramer Acetate (N = 9), Teriflunomide (N = 9), Other (N = 24), None (N = 4). The average time since diagnosis was 10.5 (±7.3) years. All patients completed the Multiple Sclerosis Performance Test (MSPT), a tablet-computer-based disability assessment tool [42] including the quality of life assessment Neuro-QoL Quality of Life in Neurological Disorders [40,43], within 3 months before study participation as part of the standard Multiple Sclerosis Partners Advancing Technology and Health Solutions (MS PATHS) clinical routine [44]. The MSPT assesses manual dexterity for both upper extremities by means of a nine whole-peg test, and also includes a self-report eight-item scale (Neuro-Qol lower extremity: Mobility). The Expanded Disability Status Scale EDSS; (EDSS); [45] and Multiple Sclerosis Severity Score (MSSS); [46] was assessed by a trained physician in the last three months prior to the study participation. The MSPT, EDSS and MSSS scores are given in Table 1.

### 2.2. Dual-Task

**Figure 1:**
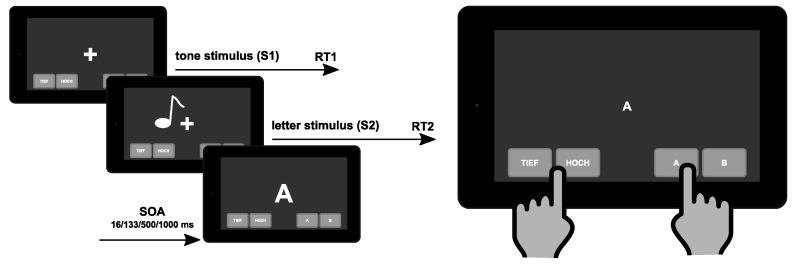
Schematical illustration of the psychological refractory period (PRP) paradigm. The tone stimulus (S1) is presented first. The letter stimulus (S2) is presented with a defined stimulus-onset asynchrony (SOA). Participants are asked to respond as soon as possible to the tone stimulus by pressing one of two buttons with their left index finger and to respond as fast as possible to the letter stimulus by pressing one of two buttons with their right index finger.

The dual-task was conducted on a Galaxy Tab A 10.5 (SM-T590) with Android 9 (Samsung Electronics Co., Ltd.) tablet computer with a 24.54 cm capacitive touchscreen, a screen resolution of 1920 × 1200 px, running on the Android (Open Handset Alliance) operating system. The dual-task was identically structured as in a previous study, first introducing this test in the cognitive assessment of MS patients [32]. The structure of the task (i.e., succession of stimuli and required responses) is shown in Figure 1. There was a “tone task” and a “letter task” [47]. Tones were delivered via headphones and were pitches of 300 or 900 Hz frequency. Each tone was presented for 200 ms. During the “letter task”, the letters “H” and “O” were used to ease discrimination of the stimuli. The dimensions of the stimuli were 1.2 × 1 cm and the stimuli were presented in the centre of the screen.

The tone task was always presented first. The time differences between the presentation of the tone stimulus and the presentation of the letter stimulus (stimulus-onset asynchrony, SOA) were varied in four steps: 16, 133, 500 and 1000 ms. Each SOA was presented 36 times. The test was divided into three blocks of 48 trials each. In each block, all possible tone–letter combinations and the different SOAs occurred equally frequently and were presented in a pseudo-randomized fashion. To respond in the task, the participants had to operate four buttons. Two of these were located in the left corner (one for a high pitch and one for a low pitch), the two in the right corner of the tablet’s touchscreen (one for letter “A” and one for letter “B”). A left-hand index finger response was performed for tones, a right index finger response for the letters. Each trial started with the presentation of a central fixation cross. The response time window was restricted to 2000 ms. If no response occurred within this period, the trial was considered a miss. In this case, the next trial started within a randomly jittered interval of 500 to 2500 ms (mean 1500 ms). If a valid response was given, the next trial started after a response stimulus interval (RSI) of 2000 ms, jittered between 1000 and 4000 ms. Participants were asked to respond as quickly and accurately as possible and to place equal emphasis on both tasks. Additionally, the participants were instructed to respond first to the tone stimulus (S1) and second to the letter stimulus (S2). Prior to the experiment, all participants completed two exercise blocks of each 12 trials.

### 2.3. Implementation of the Dual-Task as Mobile E-Health Tool

The Dual-Task, described above, was developed as a server-based progressive web application. Therefore, it can be executed on a wide range of devices, like tablet computers and smartphones. Dual-tasking based patient testing is carried out in the web browser and the data are stored and processed on the corresponding server. The application is divided into front-end and back-end. The front-end part runs in the browser of the end user on the end user’s mobile device. In the Vue.js-based interface of the front-end, the stimuli used in the task are presented. The participant can learn the functions of the interface in a tutorial. The front-end is optimized in such a way that no disturbing influences from the user interface are displayed and the participant can concentrate completely on the execution of the dual-tasking test. After completion of a test, the participant can view and evaluate the test results in a detailed table view. The front-end is connected to the back-end via a secure HTTP over TLS connection using state-of-the-art REST interfaces. This means that all data transfer between front-end and back-end is encrypted, and therefore all test results are encrypted and securely transmitted. The back-end is based on ASP.Net Core 3.1. ASP.NET is a modern and popular web-development framework for building web apps and the NET. platform and use in all common server operating systems. The back-end securely and efficiently stores test results on the web server for easy retrieval, post-processing or evaluation.

### 2.4. Statistical Analyses

Statistical analysis was conducted using Matlab 2019a (The MathWorks, Inc., Natick Massachusetts, MA, USA) and SPSS 27 (IBM Corp.). For analysis of the PRP effect, the slope of the SOA function was computed for letter stimulus RTs (slope_RT_) and accuracy of reaction (slope_Accuracy_). Reaction times (RTs) and accuracy rates were analyzed using repeated measures ANOVAs with the within-subject factor “SOA” (SOA 16 vs. SOA 133 vs. SOA 500 vs. SOA 1000). ANOVAs were conducted for the overall results, separately for block 1 to 3 and for the data of block 1 and 2 as well as block 2 and 3. Greenhouse–Geisser corrections were applied when necessary. Pair-wise comparisons are Bonferroni-corrected. For correlation analysis, Pearson’s linear correlation coefficient was computed. Cronbach’s alpha was computed as a measure of internal consistency of block 1, 2 and 3.

## 3. Results

**Figure 2:**
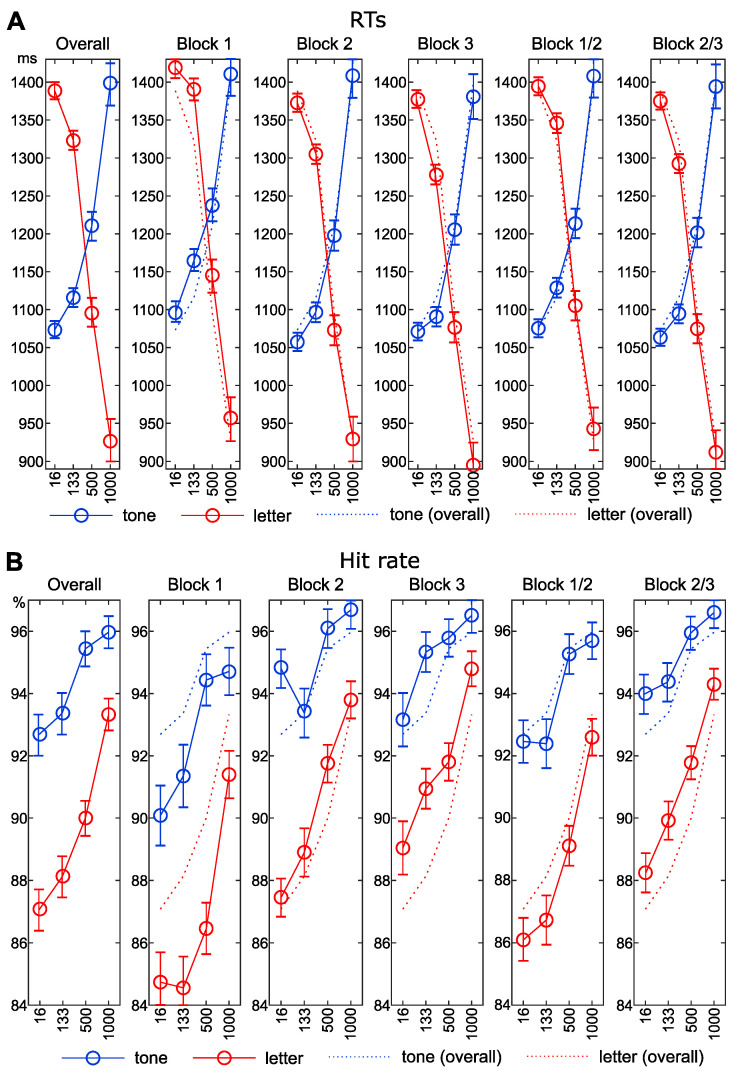
RTs (**A**) and hit rates (**B**) for the tone and letter stimulus, separately for the entire test (overall), for each block, aggregated for block 1 and 2 as well as for block 2 and 3. The *x*-axis denotes the stimulus-onset asynchrony (SOA), the *y*-axis denotes the RT in ms (**A**) or accuracy rate in % (**B**). Error bars indicate standard error of the mean (SEM). The line color denotes tone stimulus (blue) or letter stimulus (red). The dashed line indicates data from overall blocks for comparison.

Due to the focus on the PRP effect (i.e., the modulation of response selection processes triggered by the second stimulus), the analysis was limited to the RTs and the accuracy rates to the letter stimulus (S2). Mean RTs and hit rates for each block are given in Figure 2. Mean RTs, hit rates, slope_RT_ and slope_Accuracy_ function per block are given in Table 2.

### 3.1. Reaction Times

Regarding the RTs to the letter stimulus in correct trials for all blocks, i.e., all trials, the repeated measures ANOVA showed a significant main effect of SOA (F (1.69, 309.68) = 1193.04; *p* < 0.001; η^2^p = 0.87). Bonferroni-corrected pairwise comparisons showed that the RTs of all SOA differed significantly from each other (all *p* < 0.001) and significantly decreased from SOA 16 to SOA 1000.

Looking at block 1 (i.e., the initial first third of all trials), the ANOVA for RTs again yielded a significant main effect (F (2.34, 427.28) = 715.46; *p* < 0.001; η^2^p = 0.8). Similar to the overall analysis, RTs decreased with increased SOA and all SOA differed significantly from each other (*p* < 0.002).

For block 2, there was a significant main effect (F (2.011, 367.999) = 757.2; *p* < 0.001; η^2^p = 0.81). RTs of all SOA differed significantly from each other (*p* < 0.001) and decreased with longer SOA. A main effect was also found for hit rates (F (2.63, 480.5) = 16.56; *p* < 0.001; η^2^p = 0.08).

Block 3 RTs again showed a main effect (F (2.22, 405.76) = 712,46; *p* < 0.001; η^2^p = 0.8). RTs decreased with longer SOA and all SOA RTs differed significantly (*p* < 0.001).

For the RTs of block 1 and 2, i.e., the first two thirds of trials) the repeated measures ANOVA showed a main effect (F (1.82, 332.26) = 1052.06; *p* < 0.001; η^2^p = 0.85). The RTs differed between all blocks (*p* < 0.001) and longer SOAs were linked to shorter RTs.

Regarding trials of block 2 and 3, i.e., the last two thirds of trials, a main effect was observed (F (1.79, 326.9) = 1003.67; *p* < 0.001; η^2^p = 0.85). RTs decreased with larger SOA and RTs differed between all SOA (*p* < 0.001).

### 3.2. Accuracy Data

Regarding the accuracy (hit rate) to the letter stimulus in correct trials for all blocks, i.e., all trials, the ANOVA showed a significant main effect (F (2.28, 416.35) = 36.66; *p* < 0.001; η^2^p = 0.17). The number of hits differed significantly between all SOAs (*p* < 0.004), except for SOA 16 and SOA 133. Longer SOA were connected to higher hit rates.

In block 1, for hit rates, the ANOVA also showed a main effect (F (2.82; 515.42) = 21.25; *p* < 0.001; η^2^p = 0.1). Hit rates at SOA 1000 differed significantly from all other SOA (*p* < 0.001), but no significant differences were found among SOA 16, SOA 133 and SOA 500 (*p* > 0.229).

For block 2, hit rates of the different SOAs differed significantly (*p* < 0.007), except for SOA 16 and SOA 133 (*p* = 0.765), as well as SOA 500 and SOA 1000 (*p* = 0.099). Longer SOAs were associated with higher hit rates.

In block 3, the accuracy also differed significantly, as shown by a significant main effect (F (2.67; 488.17) = 19.45; *p* < 0.001; η^2^p = 0.1). Longer SOAs were connected with higher hit rates (*p* < 0.007) but no differences were found for SOA 16 and SOA 133 (*p* = 0.055), as well as for SOA 133 and SOA 500 (*p* = 1).

For the accuracy pooled across blocks 1 and 2, a significant main effected could also be found (F (2.41, 441.53) = 28.05; *p* < 0.001; η^2^p = 0.13). Significant differences were evident between all SOAs (*p* < 0.006) except for SOA16 and SOA133 (*p* = 1) and longer SOAs were associated with increased hit rates.

Regarding trials of block 2 and 3, i.e., the last two thirds of trials, the main effect was also significant (F (2.3, 421.48) = 28.1; *p* < 0.001; η^2^p = 0.13). Except for SOA 16 and SOA 133 (*p* = 0.054), hit rates differed significantly between SOA (*p* < 0.007) and longer SOAs were connected to larger hit rates.

### 3.3. Reliability Analysis

For reliability analysis, Cronbach’s α was calculated for RTs and the accuracy data, as well as the slope_RT_ and the slope_Accuracy_ to assess the internal consistency of blocks 1 to 3 (see Table 3 for the RT data and Table 4 for the accuracy data). This was done separately for the tone stimulus (S1) and the letter stimulus (S2). However, the most important parameters are these in response to the letter stimulus (S2), since these reflect SOA-dependent modulations in response selection (i.e., dual-tasking abilities and the PRP effect). For the S2 reaction time data depending on the SOA condition, as can be seen in Table 3, the internal consistency is excellent, with Cronbach’s alpha ranging between 0.92 and 0.95. For the S2 accuracy data (see Table 3), Cronbach’s alpha was also good with ranges, between 0.81 and 0.89 depending on the SOA condition. For the slope of the RT and accuracy parameters, Cronbach’s alpha was lower (cf. Table 3 and Table 4). For all examined parameters (i.e., RTs, response accuracy, and the slope of these parameters), and with only a few exceptions, Block 2 showed the highest sensitivity.

### 3.4. Correlation Analysis

**Figure 3:**
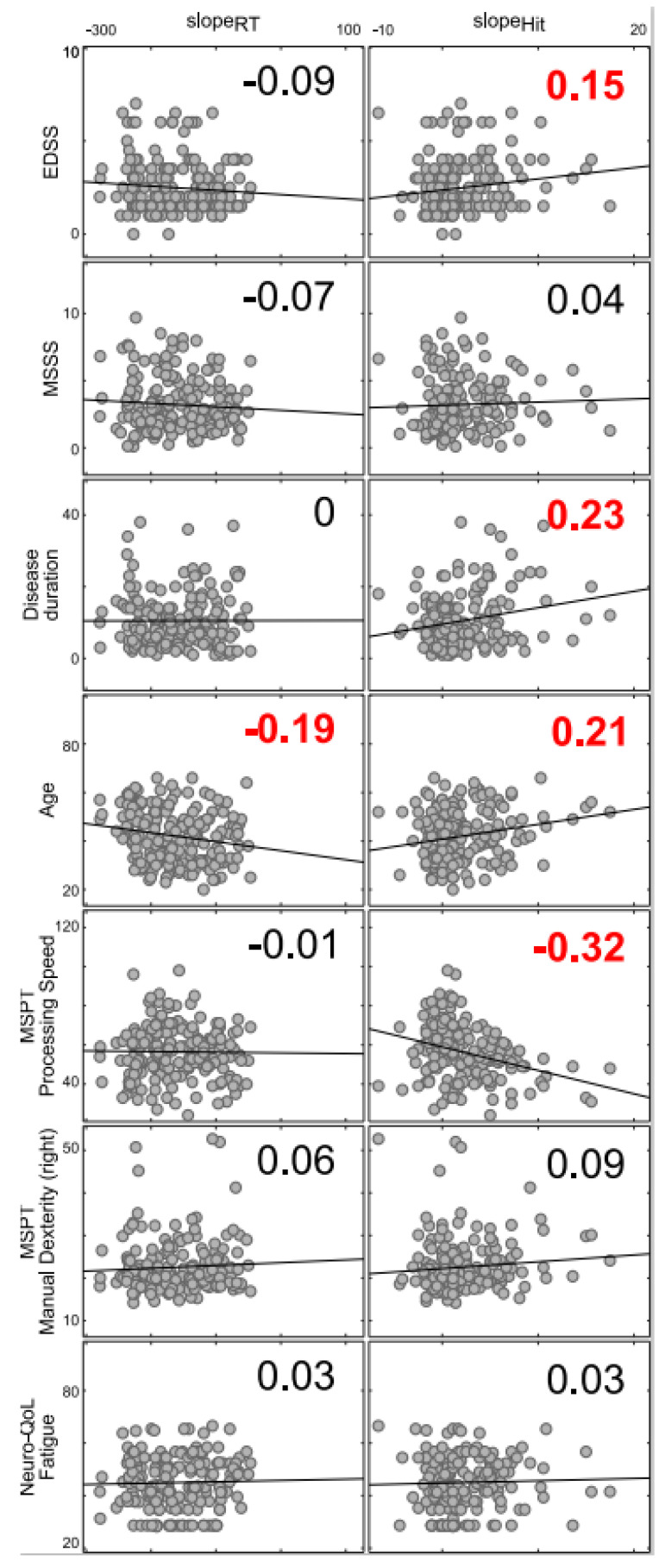
Results of correlation analysis. The numbers denote the correlation coefficient. Red font color indicates significant correlations (*p* < 0.05). EDSS: Expanded Disability Status Scale; MSSS: Multiple Sclerosis Severity Score; MSPT: Multiple Sclerosis Performance Test; Neuro-QoL: Quality of Life in Neurological Disorders.

The results of the correlation analysis of letter stimulus (S2) slope parameter for RTs and hit rates using MSPT, EDSS and MSSS data are given in Figure 3. These correlations were calculated to assess the clinical relevance/validity of the results. The slope parameter was used because this parameter provides information as to what extent response selection in dual-task situations is modulated by different levels of difficulty (i.e., SOA).

The slope_RT_ was only significantly correlated with age (r = −0.19, *p* = 0.012) and it is shown that a higher age was associated with a flatter slope_RT_. No correlations were found with clinical scales. However, regarding the slope_Accuracy_, positive correlations were found for age (r = 0.21; *p* = 0.05), EDSS (r = 0.15; *p* = 0.036) and disease duration (r = 0.23; *p* = 0.02), indicating that increased age, higher EDSS scores and longer disease durations were connected to an increased slope_Accuracy_. In contrast, a negative correlation was found for the MSPT Processing Speed/MSPT score (r = −0.32; *p* < 0.01). Higher MSPT performance was linked to a flatter slope. Generally, no correlations were obtained for the MSSS and the Neuro-QoL short-form subscale scores Anxiety, Depression and Fatigue (All r < 0.11; *p* > 0.14).

## 4. Discussion

The goal of the study was to develop a novel progressive web application dual-tasking assessment tool (e-health device) and to examine the psychometric properties of this device in terms of its reliability. Furthermore, the goal of the work was to examine how far the dual-tasking abilities examined in this task are relevant to clinical MS care by examining correlations between task performance and established clinical parameters on disease severity in MS.

Previous data using the same dual-task have already shown robust differences to healthy controls [32]. The current study was motivated by these findings and the problem that the assessment of dual-tasking abilities still reflects an unmet clinical need in the clinical care of MS patients [5]. To date, the problem was that dual-tasking assessment approaches derived from experimental psychology and cognitive neuroscience were not easily applicable in clinical practice since test administration required specific software and was not easily integrated in routine test settings (e.g., in outpatient or bedside settings) due to the long testing times and bulky devices needed to administer the test. This problem was solved in the current study by designing a progressive web application that works on any platform.

Most importantly, the data reveal that typical psychological refractory period (PRP) effects on the letter (S2) stimuli were obtained using the novel device. It is shown that reaction times were longest in the shortest SOA condition (i.e., when temporal spacing between the tone and the letter task (stimuli) was smallest). Reaction times became faster with increasing SOA time [35,36,37]. The same effect is shown for the accuracy data. Response accuracy increased from the shortest SOA condition to the longest SOA condition. Therefore, response selection accuracy in this dual task became better when the task was less taxing [38,39,40]. This pattern of findings was evident in each block of the PRP-implementation of the device. Since one major aspect that is important to consider for the clinical applicability and user acceptance of the device is testing time, this study followed the approach of building three different task blocks with identical trial numbers for each SOA condition. The fact that consistent PRP effects occurred in all three blocks shows that dual-tasking abilities were measured in the same way in all three blocks. This is a major pre-requisite to designing a test instrument that can be applied in short durations of time. This is corroborated by the reliability test assessing internal consistency, as outlined in the results section. Cronbach’s alpha for the reaction time data ranged between 0.92 and 0.95 depending on SOA condition. For the accuracy data, Cronbach’s alpha was reasonable, with ranges between 0.81 and 0.89 depending on SOA condition. The high reliability of the accuracy data is especially important for the clinical applicability. The reason for this is that motor speed (i.e., RTs) is strongly affected in MS and can bias the applicability of motor-response-dependent assessments of higher-level cognitive functions. The finding that the accuracy data turned out to be reliable in the test suggests that the accuracy parameter in tablet-based PRP implementations can be used in the neuropsychological assessment of dual-tasking abilities in MS patients. Previous findings revealed that the accuracy parameter particularly differs between MS patients and healthy controls when examining dual-tasking performance using the PRP [32]. The study by Beste et al. [32] used the same setup of stimuli (tones and letters) as well as the same timing of stimuli, as the current tablet implementation of the PRP task. All this suggests that the accuracy parameter may be used to examine dual-tasking abilities in MS. The slope of the accuracy parameter revealed a smaller reliability (i.e., Cronbach’s alpha) than the accuracy parameters for each SOA condition separately. This is an expected (mathematical) effect, since the slope parameter represents a ratio that is associated with an increase in variances in the data. However, the data also show that Block 2 showed the highest sensitivity. Each of the different three blocks in the tested implementation of the dual-task paradigm lasted for 3 min. Therefore, the data suggest that a testing time of ~6 min is required and also sufficient for a reliable estimation of dual-tasking performance in MS patients. This aspect is of high practical relevance, since it fosters the applicability of dual-tasking assessments in clinical settings. Within these 6 min testing times, all SOA conditions were administered equally often. The variation in SOA conditions allows for “adaptive testing” [32], i.e., the test applied can scale the difficulty of dual-tasking by means of different SOAs. This is an important feature for the applicability of dual-tasking assessments in longitudinal studies and to track disease progression. This makes the test suitable for adaptive testing in patients with more severe disease symptoms, especially because the accuracy parameter and the response speed parameter were shown to be reliable. It is important to stress that the task measures how reaction times/response accuracy change as a function of the SOA between two stimuli (i.e., the time between two stimuli). Since the visual stimuli were always the same, the visual aspects of the task cannot account for the for the SOA-dependent modulation of RTs (i.e., the dual-tasking effect). Although visual deficits can, in general, affect task performance, they cannot affect the parameter-indexing dual-tasking performance.

Considering the accuracy parameter (i.e., its slope), it is interesting that this parameter revealed correlations with clinically relevant parameters such as the EDSS score, disease duration and processing speed, as assessed by MSPT. EDSS score and disease duration were positively correlated with the slope. The data suggest that response selection processes in dual-tasking become more prone to variations in the difficulty selecting a response (i.e., SOA variation effect) when the EDSS score becomes higher and disease duration longer. Processing speed, as examined using the MSPT, was also correlated with the slope of the accuracy parameter. Here, a negative correlation was found, suggesting that a higher processing speed was related to a flatter slope. Hence, response selection processes in dual-tasking become less prone to variations in the difficulty to select a response when the patient has a relatively high information processing speed, as examined using the MSPT. This finding is reasonable since the PRP task has a strong speed component. All these results suggest that the tested dual-task implementation taps into clinically relevant aspects of the disease. However, it is important to note that correlation coefficients were low. This shows that the dual-task assessment is not redundant to existing clinically relevant measures of cognitive function, such as processing speed, measured using the MSPT. Rather, the data suggest that the tested e-health device complements theses existing measures by providing a reliable assessment of dual-tasking abilities in MS patients. Notably, no correlations were evident with a measure of self-reported fatigue (i.e., Neuro-QoL fatigue eight-item measure), which suggest that the test results should not be affected significantly when conducted on patients also suffering from fatigue. This is central because fatigue affects other routinely used neuropsychological assessment tools [48,49]. The Neuro-QoL fatigue scale is highly correlated with other established self-report fatigue measures, including the fatigue-subscale of the Functional Assessment of Multiple Sclerosis (FAMS) questionnaire [50] and the Patient-Reported Outcomes Measurement Information System Fatigue Item Bank (PROMIS FIB) [51]. Of note, the Neuro-QoL fatigue scale was centered and scaled using a clinical population and therefore cannot directly infer if a specific score is of clinical significance [43]. The “age” of the patients was also correlated with task performance for the accuracy and the RT data, however, a positive correlation was obtained for the accuracy data and a negative correlation was obtained for the RT data. This suggests that “age” mainly induces a speed–accuracy trade-off in dual-tasking and has no other clinical meaning.

However, some limitations should be noted. To determine the reliability of this novel tablet-computer based implementation of the PRP, the internal consistency was determined. In contrast to the retest-reliability, the internal consistency does not rely on the assumption that the construct being measured does not change over time. Assessing the internal consistency of the three consecutive blocks containing exactly the same sequence of trials helps to answers the question of both internal consistency and stability. However, stability over longer periods of time cannot be assumed for a patient group with presumably active disease progression. Future studies may, however, even in patients with a disease characterized by progression and relapses, achieve results by adopting strict inclusion/exclusion criteria (absence of relapses in the last three months and during the study), and using a short test-retest interval (e.g., few days apart). The inter-method reliability cannot be determined in this study because a direct comparison of the novel dual-task implementation with a conventional keyboard-based implementation was not conducted. However, an at least satisfactory inter-method/parallel-form reliability can be assumed, since both the novel implementation and the conventional PC-based PRP implementation reliably produce a PRP effect. Future validation steps of this dual-task implementation should include a direct comparison of both implementations and may also integrate structural MRI to examine the effects of brain structural abnormalities and their change in MS.

Handedness may influence speeded responses to the S2 stimulus, but was not assessed in this study. Although it can be assumed that RT differences due to handedness should be evenly distributed across all SOA levels, and therefore should not significantly influence the PRP effect, future validation steps should consider the effects of handedness.

The assessment of cognitive functions is becoming a cornerstone in routine clinical care and clinical trials of MS patients [1,3,5]. Especially with regard to the inclusion of cognitive tests in clinical trials, it is essential that the tests are reliable and quickly administrable. In this pilot study, we demonstrate that the test is easy to apply without need for the intense training of nurses in clinical real-world settings. The dual-task test enables an assessment using a progressive web application, which could be applied in MS centers or by the patients themselves, which makes it quickly scalable to the high case numbers in the context of clinical study situations. In addition, this clinically very relevant test [5] could be transferred to everyday clinical practice to monitor cognitive function longitudinally. We argue that the web-based technology of the tested device, including an in-built database structure, will prove especially useful in the clinical applicability of the device. Such e-health diagnostic tools are helpful to alleviate the supply shortfall in the healthcare system and to improve the care of chronically ill patients because they can present the course of the illness more comprehensively and more accurately than standard clinical visits, especially in MS [52]. Using digital tools, data collection does not increase the burden on providers or a generate significant incremental cost. Therefore, the proliferation of computerized neuropsychological assessment devices (CNADs) for screening and monitoring cognitive impairment is increasing exponentially [53]. This can support the general strategy to provide personalized MS management in which the assessment of cognitive functions using digital approaches needs to be implemented alongside immunological, genetic and MRI profiling of the individual patient [6,54].

Taken together, the presented study underlines the reliability of the developed tablet-based assessment tool for dual-tasking abilities in MS patients. The results show that it is possible to conduct a reliable assessment of multitasking abilities in about 6 min. We consider this duration acceptable for routine clinical neuropsychological assessments of dual-tasking abilities. As such, the presented assessment tool seems suitable to address a clinical need to examine dual-tasking [5]. Future studies may also evaluate this assessment tool regarding its suitability in the long-term follow up assessments of MS patients and to assess dual-tasking abilities in other neurological and psychiatric disorders.

## Figures and Tables

**Table 1:** Expanded Disability Status Scale (EDSS), Multiple Sclerosis Severity Score (MSSS) and Multiple Sclerosis Performance Test (MSPT) scores. The mean and standard deviation (SD) are given.

	Mean	SD
*EDSS*		
Total score	2.5	1.4
Visual	0.6	0.8
Brainstem	0.6	0.6
Cerebellar	0.9	0.9
Sensory	1.1	1.0
Bowel_Bladder	0.5	0.8
Cerebral	1.0	0.9
Ambulation	0.8	1.8
MSSS		
Total score	3.2	2.0
*MSPT*		
Processing Speed Test/SDMT	56.5	13.5
Low Contrast Letter Acuity Test	39.7	8.0
Manual Dexterity Test right	22.6	6.2
Manual Dexterity Test left	22.7	5.8
Walking Speed Test	5.2	2.2
*Neuro-QoL*		
Ability to Participate in Social Roles and Activities	49.8	8.0
Satisfaction with Social Roles and Activities	49.7	7.2
Depression	44.9	7.9
Emotional and Behavioral Dyscontrol	47.7	9.3
Stigma	44.4	7.9
Applied Cognition	50.7	9.5
Positive Affect and Well-Being	102.1	584.9
Fatigue	45.0	10.0
Sleep Disturbance	48.2	10.2
Lower Extremity (Mobility)	53.6	9.0
Upper Extremity (Fine Motor)	49.5	8.9

**Table 2:** Mean RTs, accuracy rates, slope_RT_ and slope_Accuracy_ (± standard error of the mean, SEM) of the letter stimulus (S2) for each stimulus-onset asynchrony (SOA) condition.

	SOA 16	SOA 133	SOA 500	SOA 1000	Slope SOA
*S2 RTs*					
Overall	1388.5 ± 14	1322.9 ± 14.5	1095.4 ± 18.9	926.5 ± 19.1	462 ± 11.2
block 1	1419 ± 14.8	139.5 ± 16.9	1145.3 ± 21	957.2 ± 2.5	461.9 ± 12.9
block 2	1372.5 ± 15.3	1304.9 ± 15.7	1072.9 ± 19.7	929.5 ± 2.4	443 ± 12.5
block 3	1377.3 ± 14.5	1277.7 ± 14.4	1076.5 ± 19.7	894.9 ± 19	482.3 ± 13.3
block 1/2	1394.3 ± 14.4	1346 ± 15.4	1105.2 ± 19.2	942.9 ± 19.8	451.5 ± 11.6
block 2/3	1375 ± 14.4	1292.6 ± 14.4	1074.6 ± 19	912 ± 19.2	463 ± 11.8
*S2 accuracy*					
Overall	87.1 ± 1.2	88.1 ± 1	90 ± 0.9	93.3 ± 0.7	−6.3 ± 0.8
block 1	84.7 ± 1.3	84.6 ± 1.3	86.5 ± 1.2	91.4 ± 0.9	−6.7 ± 1.1
block 2	87.5 ± 1.3	88.9 ± 1.1	91.8 ± 0.9	93.8 ± 0.9	−6.3 ± 1.2
block 3	89 ± 1.2	9.9 ± 1.1	91.8 ± 0.8	94.8 ± 0.8	−5.8 ± 0.9
block 1/2	86.1 ± 1.2	86.7 ± 1.1	89.1 ± 1	92.6 ± 0.8	−6.5 ± 1
block 2/3	88.2 ± 1.2	89.9 ± 1	91.8 ± 0.8	94.3 ± 0.8	−6 ± 0.9

**Table 3:** Cronbach’s α reliability analysis for the slope_RT_ and RTs in each of the different stimulus-onset asynchrony (SOA) conditions and blocks.

Tone Stimulus (S1)	Cronbach’s α	Sensitivity	α If Deleted	Part-Whole-Correction	Letter Stimulus (S2)	Cronbach’s α	Sensitivity	α If Deleted	Part-Whole-Correction
slope_RT_					slope_RT_				
block 1	0.93	0.80	0.93	−0.01	block 1	0.84	0.64	0.84	0
block 2		0.89	0.86	0.07	block 2		0.78	0.71	0.13
block 3		0.86	0.88	0.04	block 3		0.71	0.79	0.06
RTs SOA 16					RTs SOA 16				
block 1	0.91	0.80	0.91	0.01	block 1	0.94	0.85	0.93	0.01
block 2		0.86	0.84	0.07	block 2		0.91	0.89	0.05
block 3		0.82	0.87	0.04	block 3		0.88	0.91	0.03
RTs SOA 133					RTs SOA 133				
block 1	0.92	0.83	0.91	0.01	block 1	0.92	0.82	0.90	0.02
block 2		0.86	0.88	0.04	block 2		0.87	0.85	0.06
block 3		0.86	0.88	0.04	block 3		0.83	0.89	0.03
RTs SOA 500					RTs SOA 500				
block 1	0.93	0.80	0.94	−0.01	block 1	0.94	0.84	0.94	0
block 2		0.89	0.86	0.07	block 2		0.91	0.89	0.05
block 3		0.86	0.89	0.04	block 3		0.88	0.91	0.03
RTs SOA 1000					RTs SOA 1000				
block 1	0.96	0.88	0.96	0	block 1	0.95	0.87	0.95	0
block 2		0.94	0.92	0.04	block 2		0.93	0.91	0.05
block 3		0.92	0.93	0.02	block 3		0.90	0.93	0.02

**Table 4:** Cronbach’s α reliability analysis for the slope of the stimulus-onset asynchrony (SOA) accuracy rates function and accuracies in each of the different SOA conditions and blocks.

Tone Stimulus (S1)	Cronbach’s α	Sensitivity	α If Deleted	Part-Whole-Correction	Letter Stimulus (S2)	Cronbach’s α	Sensitivity	α If Deleted	Part-Whole-Correction
slope_Hit_					slope_RT_				
block 1	0	0.20	0	0	block 1	0.67	0.46	0.61	0.06
block 2		0.11	0	0	block 2		0.58	0.42	0.25
block 3		0.22	0.20	−0.20	block 3		0.42	0.65	0.02
Hits SOA 16					Hits SOA 16				
block 1	0.72	0.57	0.61	0.11	block 1	0.89	0.75	0.86	0.03
block 2		0.54	0.66	0.06	block 2		0.82	0.79	0.09
block 3		0.56	0.60	0.11	block 3		0.76	0.86	0.03
Hits SOA 133					Hits SOA 133				
block 1	0.73	0.57	0.66	0.07	block 1	0.86	0.72	0.83	0.03
block 2		0.63	0.55	0.18	block 2		0.75	0.78	0.07
block 3		0.52	0.70	0.03	block 3		0.75	0.79	0.06
Hits SOA 500					Hits SOA 500				
block 1	0.75	0.58	0.70	0.05	block 1	0.86	0.77	0.79	0.07
block 2		0.66	0.58	0.17	block 2		0.73	0.80	0.05
block 3		0.53	0.72	0.03	block 3		0.75	0.80	0.06
Hits SOA 1000					Hits SOA 1000				
block 1	0.70	0.52	0.64	0.06	block 1	0.81	0.61	0.79	0.02
block 2		0.59	0.53	0.17	block 2		0.70	0.70	0.11
block 3		0.48	0.66	0.04	block 3		0.67	0.73	0.08
